# Pediatric Inflammatory Multisystem Syndrome Temporally Related With SARS-CoV-2: Immunological Similarities With Acute Rheumatic Fever and Toxic Shock Syndrome

**DOI:** 10.3389/fped.2020.00574

**Published:** 2020-09-11

**Authors:** Danilo Buonsenso, Francesca Riitano, Piero Valentini

**Affiliations:** ^1^Department of Woman and Child Health and Public Health, Fondazione Policlinico Universitario A. Gemelli IRCCS, Rome, Italy; ^2^Istituto di Microbiologia, Università Cattolica del Sacro Cuore, Rome, Italy; ^3^Istituto di Pediatria, Università Cattolica del Sacro Cuore, Rome, Italy

**Keywords:** SARS-CoV-2, COVID-19, PIMS-TS, MIS-C, acute rheumatic fever, toxic shock syndrome

## Abstract

Several studies demonstrated that COVID-19 in children is a relatively mild disease. However, recently a more serious condition characterized by systemic inflammation with clinical or microbiological evidence of exposure to SARS-CoV-2 has been described. This syndrome is now known as either “Pediatric Inflammatory Multisystem Syndrome temporally related with COVID-19” (PIMS-TS) (1), or Multisystem Inflammatory Syndrome in Children (MIS-C) (2) and is currently considered a rare post-COVID-19 complication which, in a minority of cases, can lead to death. The signs and symptoms of PIMS-TS are largely overlapping with the for Kawasaki disease (KD) and toxic shock syndrome (TSS) and are characterized, by fever, systemic inflammation, abdominal pain and cardiac involvement. In this study, we describe clinical and immunological characteristics shared by PIMS-TS, acute rheumatic fever and TSS, in order to provide hypotheses to direct future clinical and basic research studies.

## Background

The coronavirus disease 2019 (COVID19) is the result of an infection with a new virus, the SARS-CoV-2. After its first description in China, the virus spread all over the world causing millions of cases and thousands of deaths. Interestingly, the impact on children has been relatively mild (3, 4). Compared with adults, children represent a very limited part of the infected patients and the COVID-19 was critical in a minority of children. The number of pediatric deaths related to SARS-CoV-2 is extremely low and most of the time deaths were described in children with pre-existing comorbidities (3). However, after an initial optimism regarding pediatric COVID-19, a more serious condition involving children has been described during a later phase of the COVID-19 pandemic. Researchers from Italy (5), France (6), United Kingdom (7), and the USA (1), described clusters of children with systemic inflammatory disorders without an alternative diagnosis and with clinical or microbiological evidence of exposure to SARS-CoV-2. The signs and symptoms largely overlap with the ones for Kawasaki disease (KD) and toxic shock syndrome (TSS) and are characterized, among others, by fever, abdominal pain, and cardiac involvement. This syndrome is now known as either “Pediatric Inflammatory Multisystem Syndrome temporally related with COVID-19” (PIMS- TS) (2), or Multisystem Inflammatory Syndrome in Children (MIS-C) (8) and is currently considered a rare post-COVID-19 complication that, in a minority of cases, can lead to death. Although there are still several uncertainties about the etiology of this syndrome and its immunological background, there are clinical characteristics that clearly resemble other relatively common pediatric conditions. The delay between the initial SARS-CoV-2 infection and the later development of a systemic inflammatory syndrome reminds the Acute Rheumatic Fever (ARF), while the sudden onset of severe systemic inflammation with shock reminds the Toxic Shock Syndrome (TSS) driven by well-known superantigens. In this study, we describe clinical and immunological characteristics shared by PIMS-TS, ARF, and TSS, in order to provide hypotheses to direct future clinical and basic research studies.

## PIMS-TS and Acute Rheumatic Fever (ARF)

Acute rheumatic fever (ARF) is the classic example of post-infectious autoimmune disease (9). It is an immune-mediated consequence of group A beta-hemolytic streptococcal (GAS) infection which causes pharingotonsillitis, or even a paucy or asymptomatic first infection (10). Several exposures to GAS are thought to be necessary for priming of the immune responses and the later development of ARF (11). Importantly, evidence of exposure to GAS is the prerequisites for ARF diagnosis (12).

The immune response activated by the GAS infection leads to the production of antibodies able to cross-react with antigenic epitopes shared between host and bacteria (13–16). This autoimmune process is considered cause of the main clinical manifestations of ARF: carditis is related to both cross-reactive antibodies and T cells, arthritis to immune complex deposition, chorea to antibody binding to neuronal cells, while the pathophysiology of skin lesions is less clear. In particular, a mechanism of molecular mimicry has been demonstrated between group A streptococcus antigens (Streptococcal M proteins) and host tissues (10). Antibodies against streptococci's wall GlcNAc (N-acetilglucosamina) show cross-reactivity against laminin, a protein present in extracellular matrix that surrounds heart cells and in the valves (9). Importantly, ARF develops only in children with specific genetically determined host factors (HLA-DR7 was the most frequently observed associated with the disease) (9).

Interestingly, PIMS-TS seems a post acute immunological reaction to an initial SARS-CoV-2 infection, rather than an acute infection, since most children do not have positive nasopharingeal swabs but have serological evidence of a previous infection, showing positive IgG against SARS-CoV-2 in most cases (1, 5–7). Most of these children have asymptomatic or paucy-symptomatic initial SARS-CoV-2 infection, while PIMS-TS develop after expositions to virus with a few or no symptoms during initial infection. This is hypothesized after the observation that most of these children have negative nasopharyngeal PCR test but have positive IgG tests (1, 2, 5–8). We can speculate that children' multiple exposition to SARS-CoV-2 with parents with COVID-19 can work as a priming of the immune system, as happens with GAS infection and, in genetically predisposed children, lead to PIMS-TS development. Another hypothesis is that previous infections with other coronaviruses, much more frequent in the pediatric population, may have primed the child immune system to SARS-CoV-2 virus. To date, there are no evidences to support this hypothesis, however this is a potential field for future investigations.

**Figure 1:**
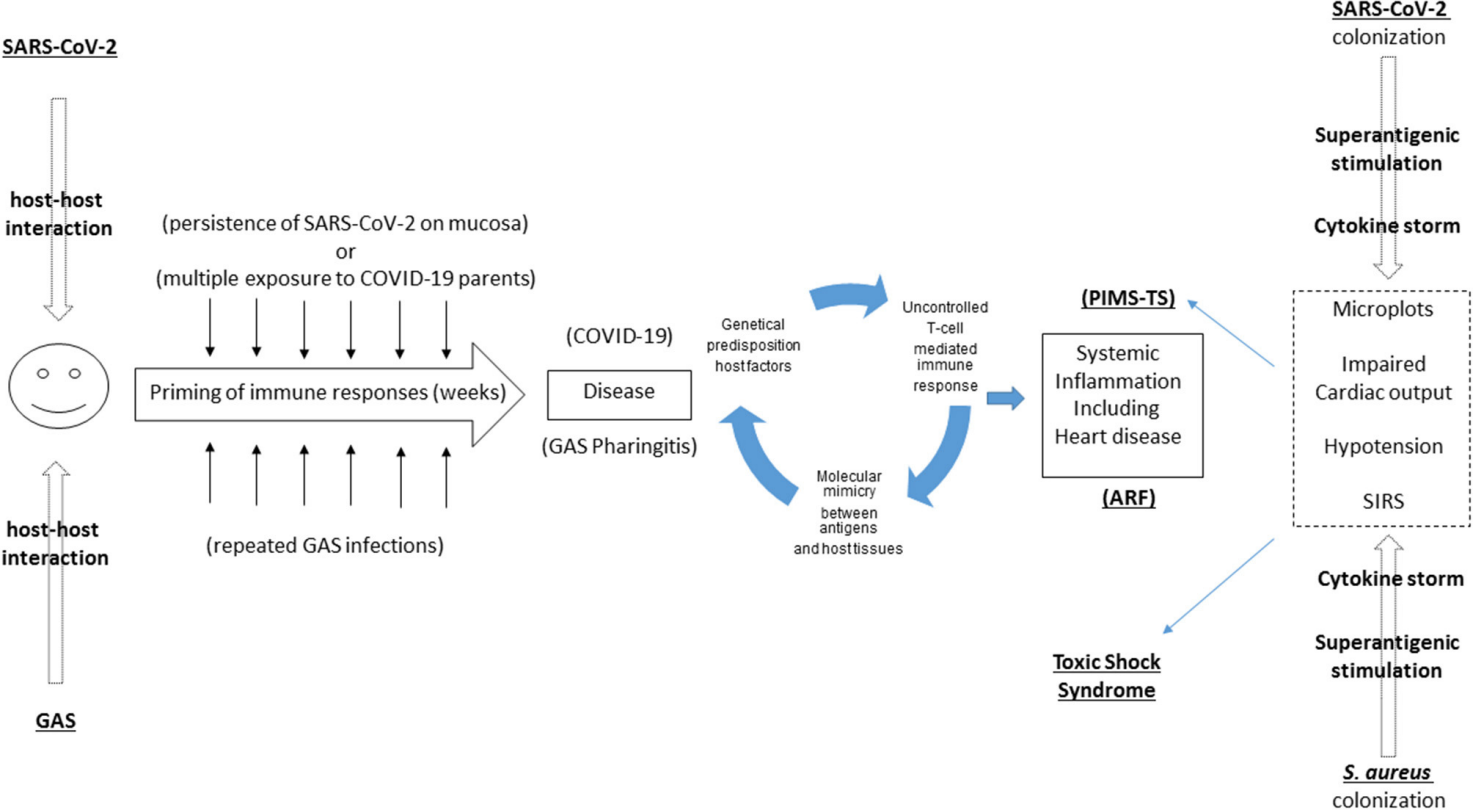
Overlaps between Pediatric Inflammatory Multisystem Syndrome temporally related with COVID-19” (PIMS-TS), Acute Rheumatic Fever (ARF), and Toxic Shock Syndrome. PIMS-TS and ARF share the delay between first infection and the subsequent development of a systemic disease. While a molecular mimicry mechanism has been documented in ARF, this is still unproven in PIMS-TS. PIMS-TS and TSS share the sudden and strong immunological storm triggered by a superantigen, recently described in SARS-CoV-2 as well.

Therefore, analyzing these two disease some analogies come up (Figure 1):

- Both of them develop after exposition to the causative agent; frequent sublinical triggers to the immune system can in both cases contribute in priming an immune response
- In both cases a clear genetic predisposition is noticed by several epidemiological studies (1, 5–7)
- Molecular mimicry plays a significant role in hearth involvement in ARF. Considering the high proportion of children with PIMS-TS developing cardiac involvement, a similar hypothesis should be tested in PIMS-TS. For example, heart involvement in PIMS-TS could be caused by the expression of ACE on heart cells, being a target of immunological responses in PIMS-TS patients (17), as documented also on a preprint paper currently under review (18).
- In both cases steroids play a primary role in controlling the inflammation and blocking the cytokine storm.

## PIMS-TS and Toxic Shock Syndrome

Toxic shock syndrome (TSS) is an acute, systemic, toxin-mediated disease, characterized by shock and multi-organ failure. It represents the most severe form of a spectrum of conditions caused by toxin-producing strains of *Staphylococcus aureus* and *Streptococcus pyogenes*. A primary role in the immuno-pathogenesis of this syndrome is played by superantigens: protein toxins can trigger excessive and non-conventional T-cell activation with consequent massive activation of other immune cells, and cytokine release. Superantigens are presented as unprocessed proteins directly to the MHC class II and to the T-cell receptor (TCR). They do not bind to the conventional peptide-binding area, primarily to the variable Vβ region on the TCR, although a small number of superantigens bind to the TCR α chain (19). The interaction of superantigen with specific TCR Vβ regions activates clonal expression of T cells and activates up to 20–30% of host T cells, whereas conventional antigen presentation activates only around 0.01% of the host T-cell population (19). Also, when superantigens bind to TCR and MHC class II, they generate a cytokine storm (19, 20). Also in the case of TSS, not all children develop this complication after *S. aureus* or GAS infection, rather a genetic predisposition is thought to be implicated. The sites at which superantigens bind to HLA class II are polymorphic (21). For example, the DRB1^*^15/DQB1^*^06haplotype is associated with protection from streptococcal TSS and reduced cytokine storm during GAS infection, whereas the DRB1^*^14/DQB1^*^05 haplotype is associated with predisposition to TSS expression (19–22).

Similarly to TSS, PIMS-TS is characterized by high grade fever, hyperinflammation, and cytokine storm (23) with multiorgan system involvement, which are highly reminiscent of TSS from both a clinical and biochemical point of view. For these reasons it is possible to speculate that also SARS-CoV-2 may present superantigenic fragments that could bind to the αβTCRs and induce an iperinflammatory response. Interestingly, Cheng et al. (24) found a motif of ~20 amino acids enclosing an insertion P_681_RRA_684_, unique to SARS-CoV-2 among beta coronaviruses, which has sequence and structure features highly similar to those of the staphylococcal enterotoxins B (SEB) toxin, through computational modeling (24). This SARS-CoV-2 protein could act as superantigen, similarly to mechanisms widely described for TSS (24). This would explain the very similar picture and the inflammatory responses described in both syndromes. As stated above, only a limited number of infected children develop PIMS-TS. It is possible that a poor initial antibody response to the virus, due to either poor exposition to the virus or to reduced affinity by SARS-CoV-2 and mucosal surfaces in children, fails to neutralize superantigen. This can lead to immune enhancement following re-exposures (25, 26). Also, a genetic predisposition can play a role, as described in TSS (19–21): specific HLA types might be more permissive of binding superantigens, and indeed preliminary reports are showing that HLA may play a role in COVID susceptibility (27). This hypothesis would be confirmed by the observation of PIMS-TS mostly in Europe and East Coast of North America and mainly Europe in children of Afro-Caribbean descent (1, 5–7), but there are not yet reported data from Asia, according to the Centers for Disease Control and Prevention and European Centers for Disease Control and Prevention (2, 8). It has been hypothesized that a mutation at D839 found in SARS-CoV-2 isolates of European Covid-19 patients enhances the binding affinity of the SAg motif to the TCR (24). This could explain the geographical skewing of PIMS-TS to areas where SARS-CoV-2 isolates of European Covid-19 patients are endemic, and identification of other strain-specific mutations may help predict where future outbreak of PIMS-TS may occur (24).

Interestingly, most of the immunomodulatory therapeutic strategies used for TSS have been shown to be effective for PIMS-TS, including intravenous immunoglobulin (IVIG) and steroids (1, 5–7). Case reports from the 1990s showed better outcome in patients with streptococcal TSS treated with IVIG (19, 28, 29). It has been suggested that IVIG can block *in-vitro* T-cell activation by Staphylococcal and streptococcal superantigens. Also, IVIG recognizes Staphylococcal Enterotoxins B (SEB) epitopes (30), and thus may function in part by neutralization of a superantigen. Given structural similarities between SEB and the S protein SAg motif of SARS-CoV-2 (24), there is potential for cross-reactivity of these immunoglobins, particularly explaining the response of PIMS-TS to IVIG.

In summary, there are analogies between PIMS-TS and TSS (Figure 1):

- Recent evidences that in both diseases superantigens can play a primary role
- In both cases a clear genetic predisposition is noticed
- In both cases IVIG play a primary role in controlling the inflammation and blocking the cytokine storm.

## Multisystem Inflammatory Syndrome in Children (MIS-C): Caution and Future Perspectives

Currently, the etiology and pathogenesis of MIS-C is not yet fully established and, to date, the epidemiologic evidences are the strongest link between MIS-C and SARS-CoV-2. To better characterize and define this link is one of the priorities of the pandemic. Since MIS-C may induce extensive immune reactions to large amounts of protein and non-protein antigens with corresponding antibody production, it is possible that patients with MIS-C can show transient cross-reactive IgG (not IgM) positive to antigens of SARS-COV-2 in the used kit. Thus, follow-up serologic examinations are needed for confirmation of SARS-CoV-2 infection.

Microbiota can also play a role in post-infectious immune-mediated diseases, such as KD. It is possible that the post-infectious uncontrolled immune responses characteristics of KD, MIS-C, and possibly ARF may be elicited by the substances derived from infected cells, including toxins, pathogen-associated molecular patterns (PAMPs), damage-associated molecular patterns (DAMPS), and pathogenic proteins and peptides described in the human microbiota (31). Thus, researchers should also address this hypothesis and evaluate if the immune responses during MIS-C may be different to those in classic KD, and infected host cells may produce different substances against different invading pathogens (microbiota). In addition, it is possible that different SARS-CoV-2 infected host cells (upper respiratory tract cells vs. cells of regional lymph nodes or lower respiratory endothelial cells) may produce different substances, which may be responsible for different clinical manifestations and incubation periods in individuals. It is known that microbiota is different in various ethnic groups and can be influenced by environment factors such as diets and antibiotics. Thus, with current knowledge, it is not possible to exclude that environmental changes caused by COVID-19 (such as closure of a school with changed diet during quarantine) can affect the transient dysbiosis of microbiota in MIS-C (32), and the subsequent immune responses, and predispose, in specific subjects, the development of post-infectious systemic immune responses.

### Limitations

The immunopathogenesis of ARF, TSS, MIS-C, and KD remains still not fully elucidated, and there are etiological substances in each disease. Thus, concepts expressed in this paper represents the summary of available evidences and the similarities between these conditions are hypotheses that still need confirmation from further studies.

## Conclusions

PIMS-TS is a newly described and still poorly characterized syndrome, particularly on an immunological point of view. The described similarities with better described pediatric syndromes can help to direct future studies. First of all, the temporal delay from the first SARS-CoV-2 infection and the development of PIMS-TS clearly reminds the correlation between GAS infection and ARF. As happened with ARF where a documentation of GAS infection is a major criterion for the diagnosis, the same could be done with PIMS-TS. In order to have a proper epidemiological and clinical picture of PIMS-TS, a clear evidence of SARS-CoV-2 exposure (either with nasopharingeal swab, presence of anti-SARS-CoV-2 IgG or clear exposition to a known adult with COVID-19) should be included as a major criterion to diagnose PIMS-TS. Current guidelines, particularly the British ones, suggest a non-specific temporal correlation with the SARS-CoV-2 pandemic other than a stronger exposure of the child with the virus. Also, the molecular mimicry described in ARF is a fascinating phenomenon that might somehow be involved in PIMS-TS, for example against ACE receptors described in heart tissue. Moreover, the description of a superantigen like protein on SARS-CoV-2 (24) is a promising discovery with potential clinical implications, particularly regarding the development of new therapeutic strategies. However, it is still unknown why asiatic children are not developing this condition, despite their well-known higher predisposition to Kawasaki disease and Kawasaki like syndrome. Specific HLA types might be more permissive of binding superantigens and predisposing to PIMS-TS development. The characterization of these HLA types can help to identify those children needing close monitoring or early anti-inflammatory treatments if infected with SARS-CoV-2. We need to highlight that PIMS-TS has been widely compared with Kawasaki Disease particularly after its first description. Even though similarities have been initially highlighted between PIMS-TS and Kawasaki Disease, recent data suggest that these conditions have several differences. Demographic features of patients with PIMS-TS, to date, are different from those of KD patient, as well clinical presentation (PIMS-TS have more frequently gastrointestinal symptoms), cardiac involvement more severe, and laboratory findings differ as well (33, 34). However, since the etiology and the immunological mechanisms leading to the development of Kawasaki Disease are far to be established, we decided to not discuss it deeply in this report. Importantly, new discoveries on PIMS-TS immunopathogenesis might be translated on ARF, Kawasaki Disease and TSS as well, therefore stimulating knowledge about the etiology and pathophysiology of all these conditions, with the benefit of a large number of children worldwide.

## Author Contributions

DB and PV conceptualized the study, collected data, wrote the draft, and final version of the study. FR was responsible for data collection. All authors wrote the final version of the study and agreed with it.

## Conflict of Interest

The authors declare that the research was conducted in the absence of any commercial or financial relationships that could be construed as a potential conflict of interest.
